# Usefulness of Plasma YKL-40 in Management of Community-Acquired Pneumonia Severity in Patients

**DOI:** 10.3390/ijms141122817

**Published:** 2013-11-19

**Authors:** Hsiang-Ling Wang, Pei-Ching Hsiao, Hsiu-Ting Tsai, Chao-Bin Yeh, Shun-Fa Yang

**Affiliations:** 1 Institute of Medicine, Chung Shan Medical University, Taichung 402, Taiwan; E-Mail: charmaine@nutc.edu.tw; 2 Department of Beauty Science, National Taichung University of Science and Technology, Taichung 402, Taiwan; 3 School of Medicine, Chung Shan Medical University, Taichung 402, Taiwan; E-Mail: cshy046@csh.org.tw; 4 Department of Internal Medicine, Chung Shan Medical University Hospital, Taichung 402, Taiwan; 5 School of Nursing, Chung Shan Medical University, Taichung 402, Taiwan; E-Mail: hsiuting@csmu.edu.tw; 6 Department of Emergency Medicine, School of Medicine, Chung Shan Medical University, Taichung 402, Taiwan; 7 Department of Emergency Medicine, Chung Shan Medical University Hospital, Taichung 402, Taiwan; 8 Department of Medical Research, Chung Shan Medical University Hospital, Taichung 402, Taiwan

**Keywords:** community-acquired pneumonia, YKL-40, severity

## Abstract

Plasma YKL-40 level has been reported as playing a significant role in community-acquired pneumonia (CAP). However, the correlation between plasma level of YKL-40 and the severity of CAP has not been reported. This study identifies the relationship between plasma level changes of the *YKL-40* gene in adult patients hospitalized with CAP. The ELISA was used to measure the plasma YKL-40 level from 61 adult CAP patients before and after antibiotic treatment and from 60 healthy controls. The plasma YKL-40 levels were significantly increased in patients with CAP compared to normal controls. Moreover, the plasma concentration of YKL-40 correlated with the severity of CAP based on the pneumonia severity index (PSI) score (*r* = 0.630, *p* < 0.001), the CURB-65 (confusion, uremia, respiratory rate, BP, age 65 years) score (*r* = 0.640, *p* < 0.001), the Acute Physiology And Chronic Health Evaluation II (APACHE II) score (*r* = 0.539, *p* < 0.001) and length of hospital stay (*r* = 0.321, *p =* 0.011), respectively. In conclusion, plasma YKL-40 may play a role in the diagnosis and clinical assessment of CAP severity, which could potentially guide the development of treatment strategies.

## 1. Introduction

Pneumonia is an inflammatory condition of the lung that involves the lung parenchyma. Community-acquired pneumonia (CAP) describes one of several diseases in which people who have not recently been hospitalized develop an infection of the lungs. CAP may be caused by bacterial, viral, fungal, or parasite agents with the complication of upper respiratory tract infection [1]. Moreover, CAP is the most frequently occurring infectious disease in the world, with high morbidity and mortality rates. In 2006, CAP was the eighth leading cause of death (along with influenza) in the United States [2]. Timely diagnosis and optimized antibiotic treatment are critical for reducing CAP morbidity and mortality. Early diagnosis and recognition of CAP severity is necessary for early goal treatment, increasing the survival rate of CAP patients [3].

YKL-40 is the first 3 *N*-terminal amino acids, and 40 denotes its molecular mass in kilodaltons (KDa). It is a heparin-, collagen-, and chitin-binding plasma glycoprotein that belong to the chitinase protein family [4–7]. YKL-40 is also a pro-inflammatory cytokine of the chitinase family 18 of glycosyl hydrolases secreted by activated human macrophages, neutrophils, and vascular smooth muscle cells [8–10]. YKL-40 is highly and positively correlated with the presence and severity of infectious diseases [11–16]. Moreover, YKL-40 is emerging as a new biomarker of severe disease activity in patients with diseases related to the degree of inflammation, pathological tissue remodeling, and ongoing fibrosis [12,17]. Ober *et al.* [18] reported that CHI3L1 is a susceptibility gene for asthma, bronchial hyperresponsiveness, and reduced lung function, and elevated circulating YKL-40 levels are a biomarker for asthma and decline in lung function. However, to the best of our knowledge, no study has investigated the prognostic value of YKL-40 in a cohort of patients with CAP. Herein, we measured plasma levels of the YKL-40 protein in a group of patients with CAP and in healthy control subjects in order to evaluate whether YKL-40 could be a useful biochemical marker to help differentiate between healthy subjects and patients with pulmonary infectious disease.

## 2. Results and Discussion

Table 1 presents a summary of the demographic and clinical characteristics. The analysis in this study is based on a sample of 121 people, and the age and the percentage of male participants was not significantly different between the CAP patients and the control group. The blood cell counts of white blood cells (WBCs) and neutrophils and C-reactive protein (CRP) levels were significantly elevated in patients with CAP before they received treatment compared with healthy controls (*p* < 0.001; Table 1) and those in patients with CAP after they received treatment (*p* < 0.001; Table 1).

**Figure 1:**
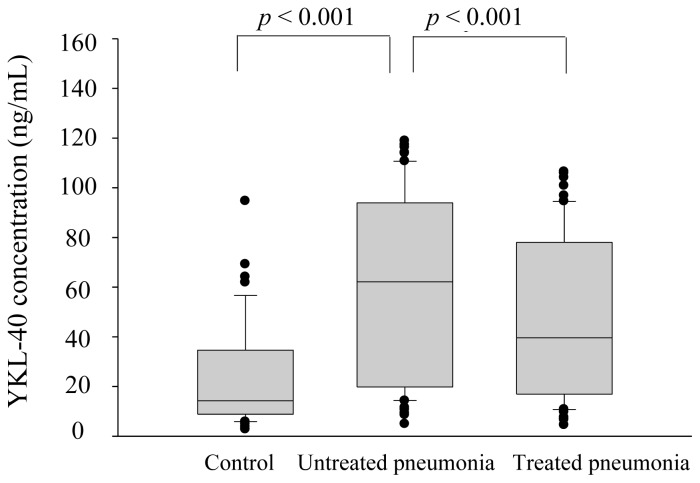
Levels of plasma YKL-40 in 61 patients with community-acquired pneumonia and 60 control patients based on ELISA analysis.

**Figure 2:**
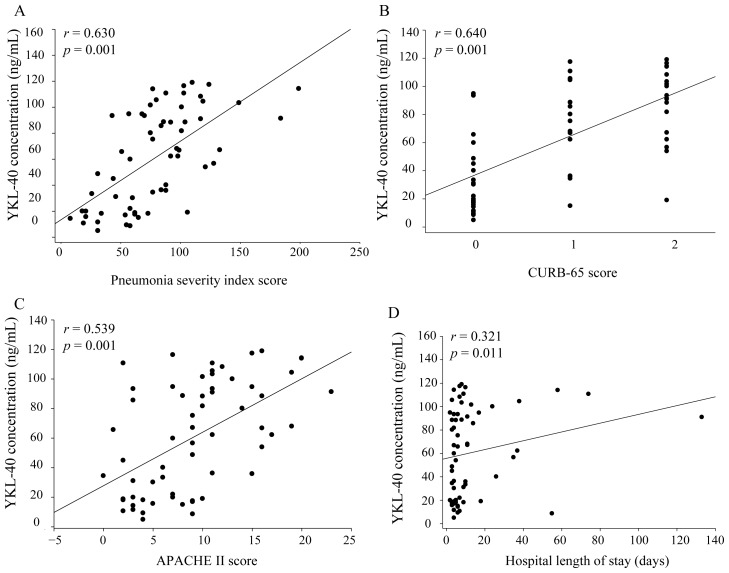
Analysis of the correlation between YKL-40 expression with PSI, CURB-65, APACHE II scores and hospital length of stay. Before treatment, CAP patients exhibited correlations among PSI, CURB-65, APACHE II scores, hospital length of stay and YKL-40 expression. There were significant correlations between YKL-40 and PSI (Spearman correlation coefficients *r* = 0.630, *p* < 0.001, *n* = 61; (**A**) CURB-65 (Spearman correlation coefficients *r* = 0.640, *p* < 0.001, *n* = 61; (**B**) APACHE II (Spearman correlation coefficients *r* = 0.539, *p* < 0.001, *n* = 61; (**C**) and hospital length of stay (Spearman correlation coefficient *r* = 0.321, *p*=0.011, *n* = 61; (**D**) in CAP patients before they received treatment.

Figure 1 shows the YKL-40 expressions of CAP patients and normal controls. There was a significantly increased YKL-40 level (*p* < 0.001) in plasma from patients with CAP before receiving treatment (61.1 ± 7.5 ng/mL) compared to healthy controls (23.1 ± 2.7 ng/mL), which significantly decreased (*p* < 0.001) after treatment (46.9 ± 4.1 ng/mL). Figure 2 shows the correlations among PSI, CURB-65, and APACHE II scores and YKL-40 in CAP patients before treatment. There were significant correlations between YKL-40 and PSI (*r* = 0.630, *p* < 0.001, *n* = 61; Figure 2A), CURB-65 (*r* = 0.640, *p* < 0.001, *n* = 61; Figure 2B), and APACHE II (*r* = 0.539, *p* < 0.001, *n* = 61; Figure 2C). Additionally, YKL-40 exhibited a significant correlation with the length of hospital stay (*r* = 0.321, *p* = 0.011) (Figure 2D). In contrast to PSI, CURB-65 and APACHE II scores, there were not significantly correlated between YKL-40 levels and WBC, neutrophils and CRP levels in the CAP patients before they received treatment (*p* = 0.510, 0.424 and 0.120, respectively).

When the cutoff level of plasma YKL-40 level was determined at 48.5 ng/mL based on ROC analysis, the sensitivity, specificity, positive predictive values and negative predictive values, as well as the likelihood ratios of positive and negative results for CAP are 59.01%, 86.67%, 81.82%, 67.5%, 4.4 and 0.5. The area under the curve of plasma YKL-40 level was 0.81 (*p* < 0.001; 95% confidence interval = 0.74–0.89).

In this study, we focus on effect size (mean difference) of YKL-40 level expression between groups. Assuming α value was set at 0.01, our sample size had at least 99% power to detect a mean difference of 20.1 based on the standard deviation was 21.07 and the alternative hypothesis was two-sided [11,19]. The results presented here show that plasma YKL-40 levels were higher in patients with CAP compared to those of healthy controls and significantly decreased in the same patients after they received antibiotic treatment. Létuvé *et al.* [14] reported that circulation and alveolar levels of YKL-40 are elevated in patients with chronic obstructive pulmonary disease (COPD) in association with disease severity. Similar to the results of this study, Nordenbaek *et al.* [6] reported that people with acute community-acquired pneumonia requiring hospitalization had increased serum YKL-40. People with acute bacterial pneumonia of different etiologies had markedly increased serum YKL-40 concentrations. Antibiotic treatment led to the normalization of serum YKL-40 within one week [6]. This implies that YKL-40 may serve as a specific serologic marker of granulocyte function at the site of tissue inflammation as a supplement to conventional acute-phase proteins [6,11]. This study also shows elevated plasma levels of YKL-40 in patients with pneumonia compared with healthy patients. The level of YKL-40 decreased significantly after treatment. Therefore, the plasma YKL-40 level can be used as a diagnostic and prognostic serologic marker of CAP.

Plasma YKL-40 level is kwon to be associated with cardiovascular disease, cancer or obstructive sleep apnea, as well as weight/body mass index, diabetes and parameters of glucose hemostasis such as glucose, insulin or c-peptide [20–22]. Unfortunately, such clinical characteristics of subjects were unavailable for this study and therefore have become the major limitation of this study.

## 3. Experimental Section

### 3.1. Subjects and Diagnosis

This study was conducted on a group of 121 people, including 61 CAP patients and 60 healthy controls, between February 2009 and December 2009. Venous blood samples from CAP patients were obtained through routine venipuncture in Chung Shan Medical University Hospital, Taichung, Taiwan, before and after antibiotic treatment. This study was approved by the Chung Shan Medical University Hospital Institutional Review Board, and each patient provided informed consent. We recorded the demographic characteristics, comorbidities, symptoms and signs of pneumonia, laboratory results, and previous antibiotic treatment of each patient upon admission. The diagnostic criteria for CAP were based on the guidelines of the Infectious Diseases Society of America (IDSA, Arlington, VA, USA)/American Thoracic Society (ATS, New York, NY, USA) [21]. The guidelines for CAP diagnosis included a typical infiltration change on chest X-ray films within one day of symptom occurrence and at least one clinical manifestation, such as cough, yellow and thick sputum, or high fever (>37.8 °C); or at least two minor criteria, including tachypnea, dyspnea, pleural pain, chest pain, confusion or disorientation, lung consolidation, or WBC counts of >1,2000 cells/μL. Exclusion criteria included outpatient status; transfer from another hospital or hospital admission within the previous 3 week; other acute conditions such as pulmonary edema, pulmonary embolism, or malignancy appearing during follow-up; pneumonia caused by tuberculosis or malignancy; severe immunocompromisation, including severe neutropenia (WBC count less than 1.0 × 10^9^ cells/L); organ or bone marrow transplant or HIV infection. In addition, subjects with cancer were also excluded. Pneumonia severity was assessed through the pneumonia severity index (PSI), Acute Physiology and Chronic Health Evaluation II (APACHE II) and CURB-65 (confusion, uremia, respiratory rate, BP, age 65 years) tests.

### 3.2. Subjects and Blood Specimen Collection

We enrolled 61 CAP patients and 60 healthy people to serve as a control group. All CAP patients were treated with empirical antimicrobial agents. We collected blood samples from all CAP patients to test the WBC, neutrophil cell count, *C*-reactive protein (CRP) concentration, and plasma concentration of YKL-40 before and after antibiotic treatment. Blood samples for the control group were also collected and tested. The blood samples were placed in tubes containing EDTA and were immediately centrifuged and stored at −80 °C. Depending on the severity of their conditions, the CAP patients were treated with effective and common antibiotics, such as cefuroxime, ceftizoxime, and clarithromycin. Table 1 presents a summary of clinical data and demographics of patients and controls.

### 3.3. Sample Size and Statistical Power

We focus on effect size (mean difference) of YKL-40 level expression between groups. Assuming α value was set at 0.01 and the ratio of healthy control and CAP patient was 1:1, our sample size had at least 99% power to detect a mean difference of 20.1 based on the standard deviation was 21.07 and the alternative hypothesis was two-sided [11,19].

### 3.4. Measurement of White Blood Cells (WBC), Neutrophil Cells, and C-Reactive Protein (CRP) Concentrations

CRP levels were measured by latex particle-enhanced immunonepherometric assay (BN Pro Spec^®^; Dade Behring Inc., Deerfield, IL, USA). Briefly, CRP monoclonal antibodies were coated in polystyrene particles, which agglutinate when mixed with CAP patients or control group samples. The CRP concentration in the sample is reflected by the intensity of scattered light in the nephelometer. For WBCs counts, total WBC counts and neutrophil counts were measured using a hematology analyzer (Sysmex XE-2100; Roche Diagnostics, Indianapolis, IN, USA).

### 3.5. Measurement of Plasma YKL-40 Level by Enzyme-Linked Immunosorbent Assay

The enzyme-linked immunosorbent assay (ELISA) was used to measure the plasma concentrations of YKL-40 in all blood samples (Quidel Corporation, San Diego, CA, USA). For each plasma sample, 20 μL was directly transferred to the microtest strip wells of the ELISA plate and subsequently incubated for 1 h at room temperature. After 4 washing steps, the detection antibody was added, and the reaction system was incubated for 1 h at room temperature. Antibody binding was detected with streptavidin-conjugated horseradish peroxidase and developed with a substrate solution. The reaction was then stopped, and optical density was determined with a microplate reader set at 450 nm. YKL-40 concentrations were quantitated by a calibration curve using a human YKL-40 standard. Each plasma sample was assayed according to the manufacturer’s instructions and the values were within the linear portion of the standard curve.

### 3.6. Statistical Analysis

Statistical analysis was performed using SPSS 15.0 statistics software (SPSS Inc., Chicago, IL, USA). All continuous variables were expressed as means ± SD. To compare between untreated patients and healthy controls, the statistical difference of demographical and clinical data was analyzed using the Mann-Whitney *U* test for continuous variables not following a parametric distribution, and the Wilcoxon signed ranks test was used to compare untreated and treated patients for categorical variables. The sensitivity, specificity, positive predictive value (PPV) and negative predictive value (NPV), tests of plasma YKL-40 for the prediction of CAP were then calculated to differentiate CAP patients from normal controls based on receiver-operating characteristic curve (ROC). Linear regression analysis revealed the correlation between YKL-40 levels and the clinical and laboratory variables of CAP patients. Statistical significance was defined as *p* < 0.05 in the two-tailed test.

## 4. Conclusions

This study assesses the association between plasma concentrations of YKL-40 and CAP. We confirmed the previous results that YKL-40 level is elevated in serum of patients with CAP. Moreover, the results of this study provide novel information regarding the plasma concentrations of YKL-40 in the assessment of CAP severity. The levels of YKL-40 concentration show a significant correlation with PSI, CURB-65, APACHE II scores and length of hospital stay. Additional studies are necessary to differentiate the stratification of CAP patients to determine the correlations of YKL-40 with the different pathogens.

## Figures and Tables

**Table 1:** The laboratory data of both controls and patients with community-acquired pneumonia (CAP) before and after they received treatment a.

Clinical Variables	Controls (*n* = 60) Median (Range)	Pretreated (*n* = 61) Median (Range)	Posttreated (*n* = 61) Median (Range)	*p* Value UT/C b	*p* Value UT/T c
Age	59.4 ± 1.5	59.5 ± 2.6	-	*p* = 0.963	-
Gender	-	-	-	-	-
Male	36 (60%)	37 (60.7%)	-	*p* = 0.941	-
Female	24 (40%)	24 (39.3%)	-	*-*	-
YKL-40 (ng/mL)	23.1 (2.7–94.6)	62.1 (4.8–118.8)	39.6 (4.5–106.4)	*p* < 0.001	*p* < 0.001
CRP (mg/dL)	0.3 (0.1–1.7)	8.6 (0.7–27.4)	0.9 (0.3–11.3)	*p* < 0.001	*p* < 0.001
WBC (/mm^3^)	5860 (3110–10190)	10890 (3560–32480)	8450 (3460–22340)	*p* < 0.001	*p* < 0.001
Neutrophils (/mm^3^)	3530 (1738–6046)	8673 (1032–29686)	5484 (1518–21155)	*p* < 0.001	*p* < 0.001
PSI score	-	79.0 ±5.9 e	-	-	-
CURB-65 score d	-	0.9 ± 0.1 e	-	-	-
APACHE II score	-	9.2 ± 0.7 e	-	-	-
Hospital length of stay (Days)	-	13.9 ± 2.7	-	-	-

Abbreviations: CRP, C-reactive protein; WBC, white blood cell; C, controls; UT, patients with CAP before they received treatment; T, patients with CAP after they received treatment; PSI, Pneumonia Severity Index; APACHE II, Acute Physiology And Chronic Health Evaluation II.

a *p* value < 0.05 was considered significant;

b The statistical difference was analyzed by Mann-Whitney U test;

c The statistical difference was analyzed by Wilcoxon signed ranks test;

d A six point score, one point each for confusion, blood urea nitrogen > 19 mg/dL, respiratory rate > 30/min, low systolic (<90 mmHg) or diastolic (<60 mmHg) blood pressure, and aged > 65 year;

e mean ± SE.
